# Meat Diet

**Published:** 1889-12-14

**Authors:** 


### THERAPEUTICS.

### MEAT DIET.

Dr. George Herschell, in an article headed "The Effect Upon the Human Body of a Diet Consisting Entirely of Lean Meat and Water," in a recent number of a contemporary, tells us that physiologists assert life cannot be sustained on such aliment. But experience seems to negative this. In this country, a diet of lean meat and water is used chiefly for the reduction of obesity, but in America, physicians are making use of the system in the treatment of dyspepsia, phthisis, ectasia of the stomach, and for the absorption of new growths. Textbooks tell us that a man must eat such an amount of lean meat to obtain the quantity of carbohydrates necessary to support him, that the digestive organs are unequal to the task for any length of time. But Dr. Herschell informs us there are now being exhibited, at the Westminster Aquarium, a family of savages who have lived all their lives on nothing but lean meat, fish, and water. Dr. Herschell suggests that the consideration of this tribe of people forces to one of two conclusions—either that the received opinions on the subject of diet are unreliable, or that it is possible so to alter the metabolic mechanism of the body by hereditary influence, as to enable the individual to exist in defiance of all the ordinary physiological laws. Laudois and Stirling, whose book is one of the most recent on physiology, say that to obtain 448 grms. of carbohydrates necessary, a man must eat 2,261 grms. of beef, and that a man is not able to maintain his metabolism in equilibrium on a purely flesh diet. If a man is to obtain 280 grms. C. from a flesh diet, he must consume, and digest, and assimilate more than two kilos. in twenty-four hours, and to this the digestive organs are unequal. But Dr. Salisbury, of New York, has continued to use a diet consisting entirely of lean meat and water for a period of several months, not only with perfect impunity, but with absolute benefit to the general health of the patient. Much of the basis on which dietary systems are formed is theoretical rather than practical, and it would appear that the subject claims more practical re-examination by physiologists.

